# Leaf Endophytes of *Populus trichocarpa* Act as Pathogens of Neighboring Plant Species

**DOI:** 10.3389/fmicb.2020.573056

**Published:** 2020-11-17

**Authors:** George Newcombe, Shannon J. Fraser, Mary Ridout, Posy E. Busby

**Affiliations:** ^1^Department of Forest, Rangeland, and Fire Sciences, College of Natural Resources, University of Idaho, Moscow, ID, United States; ^2^College of Agriculture and Life Sciences, University of Idaho Extension Washington County, Weiser, ID, United States; ^3^Department of Botany and Plant Pathology, College of Agricultural Sciences, Oregon State University, Corvallis, OR, United States

**Keywords:** fungal endophyte, plant pathogen, pathogen spillover, *Fusarium*, *Populus*, *Triticum*

## Abstract

The conventional definition of endophytes is that they do not cause disease, whereas pathogens do. Complicating this convention, however, is the poorly explored phenomenon that some microbes are endophytes in some plants but pathogens in others. Black cottonwood or poplar (*Populus trichocarpa*) and wheat (*Triticum aestivum*) are common wild and crop plants, respectively, in the Pacific Northwest USA. The former anchors wild, riparian communities, whereas the latter is an introduced domesticate of commercial importance in the region. We isolated *Fusarium culmorum –* a well-known pathogen of wheat causing both blight and rot – from the leaf of a black cottonwood tree in western Washington. The pathogenicity of this cottonwood isolate and of a wheat isolate of *F. culmorum* were compared by inoculating both cottonwood and wheat in a greenhouse experiment. We found that both the cottonwood and wheat isolates of *F. culmorum* significantly reduced the growth of wheat, whereas they had no impact on cottonwood growth. Our results demonstrate that the cottonwood isolate of *F. culmorum* is endophytic in one plant species but pathogenic in another. Using sequence-based methods, we found an additional 56 taxa in the foliar microbiome of cottonwood that matched the sequences of pathogens of other plants of the region. These sequence-based findings suggest, though they do not prove, that *P. trichocarpa* may host many additional pathogens of other plants.

## Introduction

The plant microbiome is thought to aid plants under stressful conditions to enable them to adapt to new habitats. This is, in essence, the habitat-adapted symbiosis hypothesis (Rodriguez et al., 2004, 2008). This hypothesis is at the heart of current attempts to engineer microbiome-based adaptation to climate change, and to other stresses of a rapidly changing world (Busby et al., 2017). One of the challenges in this effort is that plants can asymptomatically host pathogens of other plants (Porras-Alfaro and Bayman, 2011). A concern is thus that successful bio-engineering of one plant could lead to undesirable, non-target, spillover effects in another, via augmentation of a pathogen of the latter.

In plant microbiome studies currently, and in earlier studies, pathogens of other plants are regularly reported on the basis of the taxa that they represent. Pathogenic function is seldom, however, confirmed experimentally. For example, in a study of the endophytes of *Centaurea stoebe* (Shipunov et al., 2008), the following pathogens were reported without proof of function: *Botrytis cinerea*, the cause of gray mold of many other plants (though never of *C. stoebe* itself), *Diaporthe helianthi*, the cause of Phomopsis stem canker of sunflower, *Gibberella avenacea*, the cause of many rots, blights and declines of many crops (though again, not of *C. stoebe* itself), and many other taxa of *Fusarium* and *Alternaria* that are pathogens of plants other than *C. stoebe*. Sequence-based evidence of the pathogen that causes the “mal secco” disease of citrus was even found in achenes of *C. stoebe*, although inoculations were never performed to confirm function (Shipunov et al., 2008; Migheli et al., 2009). In a study of endophytes of *Bromus tectorum, Fusarium oxysporum* was reported (Baynes et al., 2012). *Fusarium oxysporum* causes vascular wilts of a very wide range of plants, as well as blights, rots and damping off (Farr and Rossman, 2020), but the endophytic isolate in *B. tectorum* might possibly have been non-pathogenic. Similarly, species of *Geniculosporium* and *Xylaria*, pathogenic to various plants, were found as endophytes in *Abies*, or fir, trees (Carroll and Carroll, 1978). Further examples from other woody plants have been summarized in a study of white pine endophytes that often resembled pathogens of other plants (Ganley et al., 2004). In all of these studies, pathogenic function was suggested by the identification of isolates and/or sequences representative of pathogenic taxa of other plants; function was not, however, proven via inoculation assay in these studies.

In contrast, in studies focused on invaded plant communities, spillover of a pathogen from one host to another has been functionally demonstrated. For example, *Alternaria* spp., isolated from the reservoir host, *Schedonorus arundinaceus* (tall fescue, a highly invasive grass species in North America), could be inoculated into co-occurring grasses in which these fungi caused disease symptoms and decreased biomass (Wilson et al., 2014). Pathogen spillover from a reservoir host to other plants can also operate within seed banks in plant communities invaded by *Bromus tectorum* (Beckstead et al., 2010). Pathogen spillover could be ecologically common and important in natural plant communities, invaded or not, but a shortage of studies hampers generalization.

In a third area of research, on pathogens shared by crop and weedy plants, there are examples of pathogen movement from weed to crop, and its epidemiological significance (Wisler and Norris, 2005). Crop rotation to reduce pathogen inoculum can even fail because weedy plants host the pathogen when the crop is absent. For instance, weedy *Chenopodium album* can host *Verticillium albo-atrum* that can then re-infect alfalfa when it is again grown in a given field (Busch and Smith, 1982). Rust fungi can be hosted by wild oats and then infect domesticated oats (Burdon et al., 1983). Movement of pathogens in the other direction (i.e., from crop plants to weeds or to wild plants) has been little studied (Blitzer et al., 2012), which is attributed to little study generally of natural plant pathosystems (Power and Mitchell, 2004).

A recent, molecular field study of foliar fungi of *Populus trichocarpa* revealed taxa that are putative pathogens of other plants (Barge et al., 2019). *P. trichocarpa* is the wild black cottonwood tree of riparian communities in the Pacific Northwest. It is frequently found in close proximity to wheat and other crops of the region. Here, we summarize the pathogens of other plants that were found in the sequence-based study (Barge et al., 2019), and report on an experiment testing the pathogenicity of *Fusarium culmorum* isolated from *P. trichocarpa* on both wheat and cottonwood. *Fusarium culmorum* is a well-known pathogen of wheat, that has not been reported as a pathogen of *P. trichocarpa*. However, *F. culmorum* has been reported as a mutualist in dunegrass of the region (Rodriguez et al., 2008). We therefore expected that our assay with a cottonwood isolate could reveal positive, negative, or neutral effects on growth of cottonwood and wheat.

## Materials and Methods

### Greenhouse Inoculation Assay

We selected one putative pathogen for a pathogenicity experiment: *Fusarium culmorum*. An isolate of *F. culmorum* collected from asymptomatic leaves of *P. trichocarpa* in a previous study (SNO-11, Busby et al., 2016) produced a colony resembling a wheat isolate of *Fusarium culmorum*, a known pathogen of wheat. We identified the isolate on the basis of micro-morphology as *Fusarium*. To obtain a species-level identification we extracted DNA from the isolate, then amplified and sequenced the full ITS region. The Sanger sequence of the full ITS region for this isolate is archived in the NCBI genbank (accession number MN154167). This particular cottonwood, or poplar isolate, *P. trichocarpa* (FCP) was then used in the inoculation experiment below to test the hypothesis of endophytes as functional pathogens of other plants. For comparative purposes we used a wheat isolate of *F. culmorum* (FCW) from a previous study (Ridout and Newcombe, 2016).

Cultures of each strain were grown on 4% potato dextrose agar (PDA) for 2 weeks. At 2 weeks plates were flushed with sterile distilled water (SDW) and conidia were loosened with a sterile, bent glass rod. The solution was homogenized with a tissue macerator (Tissue Tearor™, BioSpec Products, Bartlesville, OK, USA) and brought to volume with SDW. Conidial concentrations of the FCW and FCP suspensions were adjusted to 10^6^ conidia per milliliter using a Neubauer hemacytometer.

Seeds of *P. trichocarpa* (collected from a wild tree growing in Moscow, ID) and *T. aestivum* (University of Idaho line 306 UI-SRG, Lot: 1209 Moscow HRWW5, class hard red winter) were sown into soilless mix (Sunshine Professional Growing Mix #1, Sun Gro Horticulture, Sacramento, CA) in four-inch pots. Seeds were germinated and seedlings grown at a diurnal temperature cycle of 18°C/15°C with a 16-h day length. Ninety pots were sown for each species. Seedlings were grown in the greenhouse for 12 days prior to inoculation.

We inoculated *T. aestivum* and *P. trichocarpa* with the *F. culmorum* isolated from *P. trichocarpa* (FCP) and a pathogenic strain of *F. culmorum* (FCW) known to cause crown rot in *T. aestivum* (Washington State University/ USDA ARS, Pullman WA: cereal pathogen collections). Twelve days following sowing, 30 seedlings each of *P. trichocarpa* and *T. aestivum* were inoculated at the crown with either 10 mL of FCP suspension, 10 mL of FCW suspension, or 10 mL of SDW (sterile distilled water) for a negative control. The plants were kept in the greenhouse for 47 days while disease developed. Visual analyses were made to determine disease presence or absence in inoculated seedlings. *T. aestivum* seedlings were then harvested by removing the above-ground vegetation at the crown; *F. culmorum* causes crown rot and thus affects above-ground biomass. This biomass was then dried in an oven at 60°C for roughly 48 h, although drying time varied somewhat. Above-ground biomass of *P. trichocarpa* seedlings was harvested 4 days later and dried at 60°C for 70 h. Dry biomass was determined for all the seedlings.

### Molecular Field Survey

We used ITS sequence data from a previously published molecular field survey of *Populus trichocarpa* foliar fungi across ten watersheds in the Pacific Northwest, USA (Busby et al., 2016) to identify fungal taxa likely to cause disease in other plants. A complete description of this molecular field survey can be found in Busby et al. (2016). In brief, we sampled leaves from six trees in 10 populations. Leaves were surface sterilized and lyophilized prior to DNA extraction. We used a modified version of the primer set ITS1F and ITS2 for sequencing on the Illumina MiSeq platform (Illumina, San Diego, CA, USA) (Smith and Peay, 2014). PCR products were cleaned using the Agencourt Ampure XP kit (Beckman Coulter, Brea, CA, USA), quantified using the Qubit hs-DS-DNA kit (Invitrogen, Carlsbad, CA, USA) on a Tecan Infinite F200 Pro plate reader (Tecan, Morrisville, NC, USA) (285 nm excitation and 530 nm emission), then pooled at equimolar concentrations prior to 250-bp paired-end sequencing using Illumina MiSeq. Raw sequence data are deposited in NCBI's Short Read Archive (accession no. SRP064132, http://www.ncbi.nlm.nih.gov/books/NBK47529/).

Both QIIME (Caporaso et al., 2010) and UPARSE (Edgar, 2013) were used to process the sequence data. Forward and reverse reads were paired with USEARCH v.7.0.1001, and discarded if they contained > 0.25 expected errors. High-quality sequences were grouped into operational taxonomic units (OTUs) in USEARCH using UPARSE-OTU and UPARSE OTU algorithms at 97% similarity. Taxonomy was assigned using the BLAST algorithm in QIIME, which uses the UNITE fungal ITS database. Additionally, the 500 most abundant OTUs were checked using BLAST searches against the NCBI GenBank. In total, 968 fungal taxa were identified. For this study, we identified putative pathogens in the dataset by searching for all taxa in the USDA SMML databases (Farr and Rossman, 2020). We calculated the proportional abundance of each putative pathogen within a sample by dividing the number of pathogen reads in the sample by the number of total reads in the sample. We additionally calculated the mean proportional abundance of each putative pathogen for each tree population.

### Statistical Analysis

All analyses were performed in RStudio v.3.6.3 (RStudio Team 2015). We used a one-way analysis of variance to compare dry weights among the treatment groups in both *T. aestivum* and *P. trichocarpa*.

## Results and Discussion

### Comparison of Cottonwood and Wheat Isolates of Fusarium Culmorum

**Figure 1:**
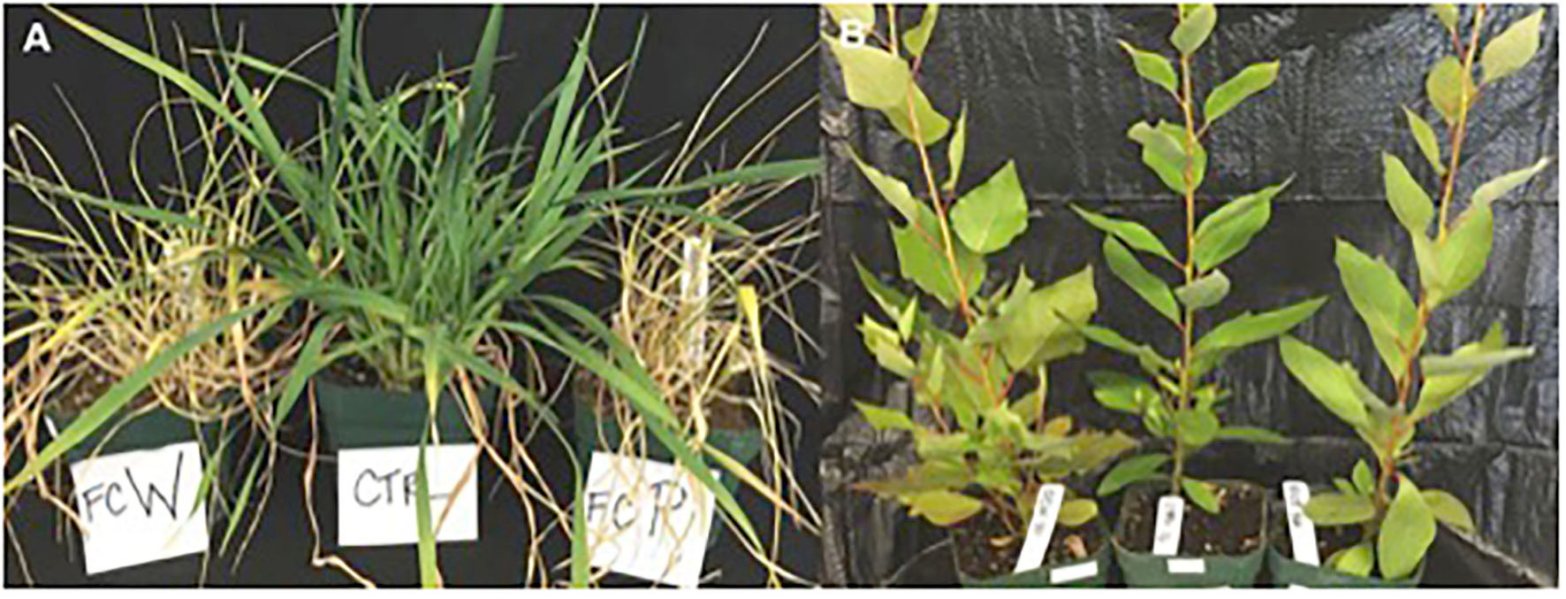
*Fusarium culmorum* isolates caused disease in wheat **(A)** but not in poplars **(B)**. Wheat and poplars were inoculated with an isolate of *F. culmorum* from wheat (FCW) and from *P. trichocarpa* (FCP), or with sterile water for the control (CTRL).

**Figure 2:**
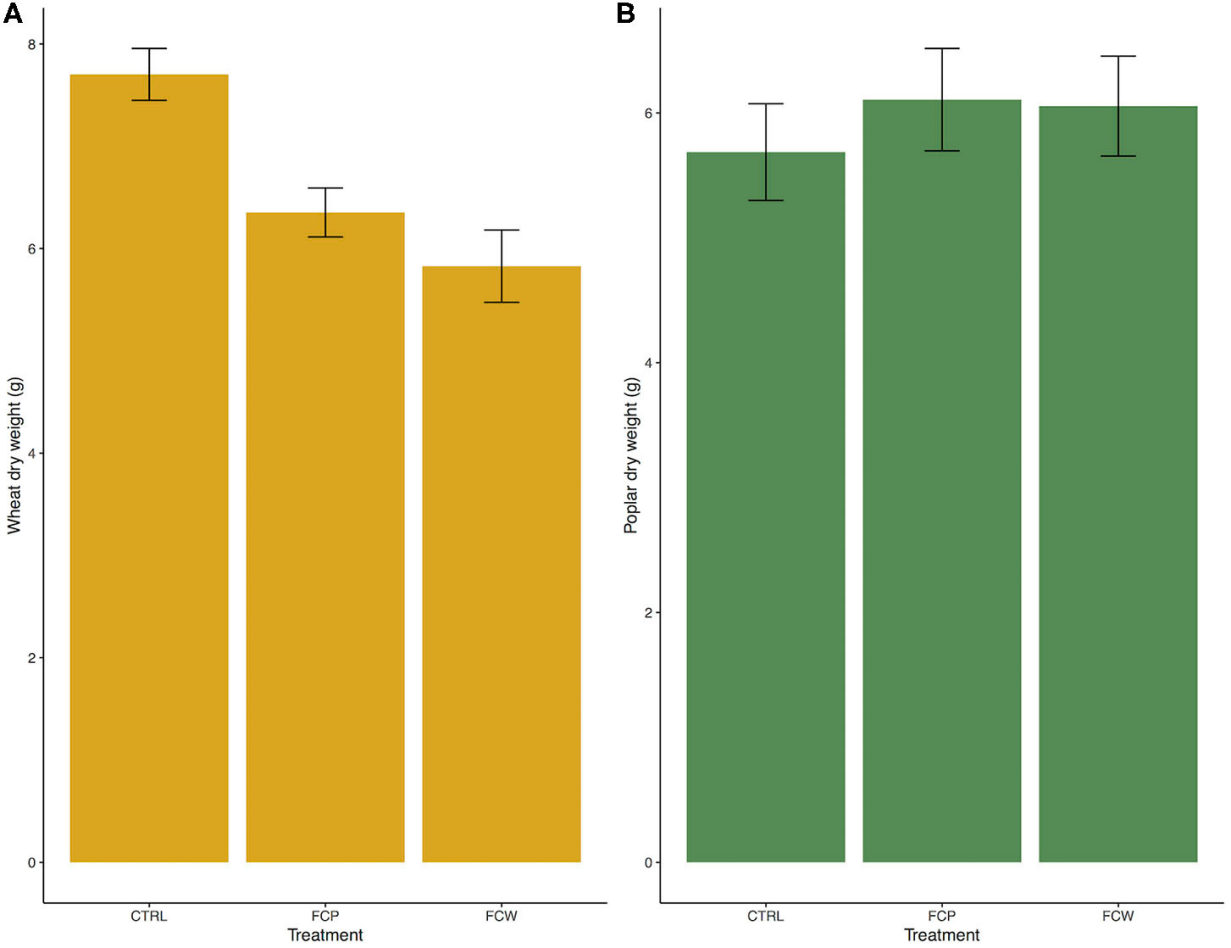
*Fusarium culmorum* isolates reduced biomass in wheat **(A)** but not in poplars **(B)**. FCW: isolate of *F. culmorum* from wheat. FCP: isolate of *F. culmorum* from *P. trichocarpa*. CTRL: sterile water control. Bars are standard error.

Both isolates of *F. culmorum –* from cottonwood and wheat – caused visual symptoms of disease (Figure 1) and a reduction in above-ground biomass for wheat; neither symptoms nor a reduction in biomass were seen in cottonwood (Figure 2). Ten days after the inoculation, wheat leaves became symptomatic. The first necrotic lesions appeared on the twelfth day post-inoculation. The uninoculated wheat controls remained asymptomatic throughout the experiment (Figure 1). After destructive sampling, the mean mass of the control plants was 7.70 grams, compared to 6.22 and 6.35 g, respectively (*F* = 11.13, *p* = 4.84 × 10^−5^; Figure 2). Thus, both isolates were associated with an ~20% reduction in wheat biomass. In contrast, both isolates of *F. culmorum –* from cottonwood and wheat – caused no visible symptoms of disease (Figure 1) nor impacted cottonwood biomass (*F* = 0.33, *p* = 0.72; Figure 2).

The USDA SMML Fungal Database shows that *F. culmorum* has previously been reported from 134 plant species, in 22 families (Farr and Rossman, 2020). It is most common as a pathogen of plants of the grass family, Poaceae, but it is common in plants of other families as an endophyte. This report is the first to mention any species of *Populus* and even its family, Salicaceae, as hosts of endophytic *F. culmorum*. The 23 families in which *F. culmorum* has now been reported are the following: Amaranthaceae, Anacardiaceae, Apiaceae, Asparagaceae, Asteraceae, Brassicaceae, Caryophyllaceae, Chenopodiaceae, Convolvulaceae, Cucurbitaceae, Cyperaceae, Ericaceae, Fabaceae, Fagaceae, Lamiaceae, Liliaceae, Linaceae, Malvaceae, Pinaceae, Poaceae, Polygonaceae, Salicaceae, and Solanaceae.

Many pathogens like *F. culmorum* are pathogenic in only some of the plant species from which they have been reported. In others they are present as endophytes or even as mutualists (Rodriguez and Redman, 2008; Rodriguez et al., 2008). In some, such as Washington's coastal dunegrass (*Leymus mollis*), *F. culmorum* is a mutualist that improves both salt and drought tolerance. With unequal effects on competing species of plants, microbes like *F. culmorum* could be important drivers of “apparent competition” (Orrock and Witter, 2010; Cobey and Lipsitch, 2013). This occurs when one competitor is favored by hosting a pathogen of the other.

### ITS sequencing of Fungal Endophytes in Cottonwood Leaves

We identified 56 sequence-based taxa in the foliar microbiome of cottonwood that are pathogens of other plants of the region (Table 1), but that are not known to cause disease in *P. trichocarpa* (Newcombe, 1996). *Cryptodiaporthe pulchella*, which is a pathogen of a few species of *Populus* and *Salix*, has not been recorded as a pathogen of *P. trichocarpa* (Farr and Rossman, 2020). Similarly, *Knufia cryptophialidica* affects *P. tremuloides* but not *P. trichocarpa* (Farr and Rossman, 2020). Some pathogens of *P. trichocarpa* were found as foliar endophytes in our earlier studies, and those have been reported and discussed elsewhere (Busby et al., 2016; Barge et al., 2019).

**Table 1:** High-throughput sequencing of leaves of *Populus trichocarpa* indicated GenBank- and USDA SMML-reported pathogens of agricultural (A) or non-agricultural plants (NA), their diseases, locations, accession numbers, and % of total sequences in the dataset.

**Pathogen**	**Host plant**	**A or NA**	**Disease**	**Location**	**Pathogen's GenBank accession number**	**% of total seqs**
*Ramularia vizellae*	*Brassica* crops	A	Leaf spot	The Netherlands	EU019285	1.8
*Monilinia sp*.	*Malus*	A	Fruit rot	Japan	AB693917	1.4
*Neofabraea malicortis*	*Malus* and *Pyrus*	A	Stem canker	The Netherlands	AF141161	<1
*Microcyclospora tardicrescens*	*Malus domestica*	A	Sooty blotch	Slovenia	GU570541	<1
*Phoma macrostoma*	*Malus domestica*	A	Fruit rot	Switzerland	HQ166389	<1
*Phaeosphaeria pontiformis*	*Triticum aestivum*	A	Leaf blight	Sweden	KC989090	<1
*Phoma macrostoma*	*Lens esculenta*	A	Bioherbicide	Canada	DQ474091	<1
*Cladosporium cladosporioides*	*Vitis vinifera*	A	Fruit rot	Chile	EU622927	<1
*Diaporthe eres*	*Vitis vinifera*	A	Dieback	California, USA	KF017914	<1
*Microcyclospora pomicola*	*Malus domestica*	A	Sooty blotch	Germany	GU570539.1	<1
*Diaporthe cf. nobilis*	*Malus pumila*	A	Dieback	New Zealand	KC343149.1	<1
*Gnomoniopsis idaeicola*	*Actinidia deliciosa*	A	Canker	France	KT692597	<1
*Devriesia pseudoamericana*	*Malus domestica*	A	Sooty blotch	Germany	GU570527	<1
*Phaeomoniella zymoides*	*Prunus salicina*	A	Wood necrosis	South Africa	GQ154600	<1
*Pyrenophora tritici-repentis*	*Triticum*	A	Tan spot	Japan	AM887495	<1
*Ramularia pratensis*	*Rumex crispus*	NA	Leaf spot	South Korea	KF251223	3.7
*Botrytis cinereal*	*Picea abies*	NA	Root rot	Canada	KF859924	1.7
*Ramularia eucalypti*	*Eucalyptus*	NA	Leaf spot	Australia	EF394862	1
*Fusarium proliferatum*	*Pinus*	NA	Pitch canker	The Netherlands	KM231816	<1
*Fusarium avenaceum*	*Phragmites australis*	NA	Leaf blight	New Jersey, USA	KT827258	<1
*Ramularia eucalypti*	*Corymbia grandifolia*	NA	Leaf spot	Italy	EF394861	<1
*Colletotrichum phormii*	*Phormium*	NA	Leaf blight	New Zealand	DQ286142.1	<1
*Sydowia polyspora*	*Pinus mugo*	NA	Needle blight	Lithuania	GQ412724	<1
*Elytroderma deformans*	*Pinus ponderosa*	NA	Needle blight	Montana, USA	AF203469	<1
*Ramularia eucalypti*	*Eucalyptus*	NA	Leaf spot	The Netherlands	KF251221	<1
*Neostagonospora caricis*	*Carex*	NA	Leaf blight	The Netherlands	KF251163	<1
*Taphrina carpini*	*Carpinus betulus*	NA	Witches' broom	Slovakia	AF492085	<1
*Curvularia trifolii*	*Leucospermum*	NA	Leaf spot	Australia	JN712459	<1
*Cryptodiaporthe pulchella*	*Salix lucida*	NA	Dieback	Maryland, USA	GU367061	<1
*Devriesia fraseriae*	*Melaleuca*	NA	Leaf spot	The Netherlands	HQ599602	<1
*Toxicocladosporium strelitziae*	*Strelitzia reginae*	NA	Floral lesions	South Africa	JX069874	<1
*Pilidium acerinum*	*Aesculus hippocastanum*	NA	Leaf blight	The Netherlands	NR_119500	<1
*Xenostigmina zilleri*	*Acer macrophyllum*	NA	Leaf spot	Canada	FJ839639	<1
*Mycosphaerella fragariae*	*Platanus*	NA	Leaf blight	South Korea	GU214691	<1
*Ilyonectria radicicola*	*Pinus sylvestris*	NA	Root rot	Sweden	KF156312	<1
*Taphrina alni*	*Alnus incana*	NA	Tongues on female catkins	Austria	AF492076	<1
*Taphrina communis*	*Prunusamericana*	NA	Plum pockets and leaf curl	USA	AF492086	<1
*Neosetophoma samarorum*	*Urtica dioica*	NA	Fruit rot	The Netherlands	KF251162	<1
*Ciborinia camelliae*	*Hepatica*	NA	Root rot	Japan	AB516659	<1
*Phoma sp*.	*Rosa rugosa*	NA	Root rot	Lithuania	KF646102	<1
*Boeremia exigua var. heteromorpha*	*Nerium oleander*	NA	Leaf spot & dieback	United Kingdom	JX467690	<1
*Drechslera dematioidea*	*Poaceae* grasses	NA	Leaf spot	British Columbia, Canada	JN712466	<1
*Diplodina microsperma*	*Protea*	NA	Leaf spot	New Zealand	JN712461	<1
*Diaporthe viticola*	*Fraxinus excelsior*	NA	Leaf spot	The Netherlands	KC343230	<1
*Phoma herbarum*	*Rosa multiflora*	NA	Leaf spot	The Netherlands	KF251212.1	<1
*Knufia cryptophialidica*	*Populus balsamifera*	NA	Stem canker	Alberta, Canada	JN040501.1	<1
*Plectosphaerella sp*.	*Alnus glutinosa*	NA	Stem canker	Latvia	JF340251.1	<1
*Plagiostoma barriae*	*Acer macrophyllum*	NA	Anthracnose	Washington, USA	EU254997.1	<1
*Strumella* sp.	*Alnus incana*	NA	Stem canker	Latvia	GU062276	<1
*Taphrina weisneri*	*Prunus fruticosa*	NA	Witches' broom and leaf curl	Portugal	AF492126.1	<1
*Rhizosphaera pseudotsugae*	*Pseudotsuga menziesii var. menziesii*	NA	Needle cast	Germany	EU700369	<1
*Diaporthe sp*.	*Actinidia*	NA	Leaf spot	New Zealand	KC145848	<1
*Teratosphaeria knoxdaviesii*	*Protea*	NA	Leaf spot	South Africa	EU707866.1	<1
*Catenulostroma hermanusense*	*Phaenocoma prolifera*	NA	Leaf bract lesions	South Africa	JF499833	<1
*Phaeosphaeria nodorum*	*Lolium perenne*	NA	Blotch	Denmark	KF251177	<1
*Septoria cretae*	*Nerium oleander*	NA	Leaf spot	Greece	KF251233.1	<1

Fifteen pathogens of agricultural or cultivated plants were found to varying extent in leaves of *P. trichocarpa* sampled in the Pacific Northwest (Table 1). These 15 fungal taxa identified by sequence homology with GenBank accessions were associated with many regionally important plants. *Malus* (apple) stood out, as 7 of the 15 pathogens were associated with this leading orchard crop of the region. Three wheat pathogens (i.e., *Pyrenophora tritici-repentis, Phaeosphaeria pontiformis, Phaeosphaeria nodorum*) were found in addition to the cultured and pathogenicity-tested isolate of *F. culmorum*. Another three taxa of fungal pathogens were found that have been associated with damage to cherry and grape, which are also regionally important crops. It is important to note that the agricultural host plants listed in Table 1 represent only a fraction of the broad host ranges of this group of 15 pathogen taxa. For example, *Phoma macrostoma* is potentially pathogenic to all dicots (Bailey et al., 2011), and as such this fungus has been of interest as a bioherbicide that could be used with monocot crops.

Among the 41 pathogens of non-agricultural plants, there were some with very broad host ranges (e.g., *Botrytis cinerea*) and others that were very host-specific (Table 1). *Elytroderma deformans* is an example of the latter as it only causes disease of species of *Pinus* subgenus *Pinus*, otherwise known as the “hard” or diploxylon pines. *Elytroderma deformans* is also limited in its distribution in North America, and its presence as an endophyte has been reported only in *Pinus* subgenus *Strobus* (Ganley et al., 2004). Thus, this report is the first for *E. deformans* in an angiosperm tree. A second example was found in the four taxa of *Taphrina* that are similarly host-specific pathogens. They parasitize species of *Prunus, Alnus*, and *Carpinus*. A third example, *Ciborinia camelliae*, specifically affects flowers of species of *Camellia* (Farr and Rossman, 2020). The most abundant pathogen was one that most closely matched a GenBank accession of *Ramularia pratensis* found on *Rumex crispus* in Korea. It is a specialized pathogen of leaves of species in only three genera, including *Rumex*, of the Polygonaceae. These genera and *Ramularia pratensis* are now found around the world where these plants have been introduced (Farr and Rossman, 2020).

In some cases the mean proportional abundance of a putative pathogen in the ITS dataset was consistent with expectations based on the abundance and distribution of the pathogen's preferred host. For example, *F. proliferatum* was more abundant in the two Idaho sites where its host, *Pinus ponderosa* (Ocamb et al., 2002), is dominant. Similarly, *R. vizellae* was more abundant in the western Washington sites where *Brassica* seed production occurs (Du Toit, 2004).

The putative pathogens of other plants, revealed here by ITS metabarcoding, would need to be tested for pathogenicity, as we have done with *F. culmorum*. If confirmed as pathogens, they could be either unspecialized and broad-range like *F. culmorum*, or highly host-specific. Host specificity varies considerably among the fungal pathogens of *Populus* (Newcombe, 1996) and among plant pathogens generally. Host ranges tend to be broader when they are compiled from identifications of pathogens and narrower when investigated via functional assay (Newcombe, 2003; Benítez et al., 2013; Sarmiento et al., 2017). However, we have not explored the transmissibility of the 56 pathogens, nor even that of *F. culmorum*, since we used artificial inoculation to prove the latter's pathogenicity toward wheat. Would cottonwood seedlings infected with *Ramularia pratensis* transmit their infection in nature to competing species of *Rumex, Rheum* or *Oxyria*? Would the competitive balance be tipped due to cottonwood's tolerance and the susceptibility of the others? Further studies are needed to explore how hosting pathogens of neighboring plants contributes to apparent competition.

## Data Availability Statement

The datasets presented in this study can be found in online repositories. The names of the repository/repositories and accession number(s) can be found at: https://www.ncbi.nlm.nih.gov/genbank/, MN154167, in NCBI's Short Read Archive (accession no. SRP064132, http://www.ncbi.nlm.nih.gov/books/NBK47529/).

## Author Contributions

GN and PB conceived of the idea for the study and wrote the manuscript with input from SF and MR. SF and MR conducted experiments. PB analyzed the data. All authors contributed to the article and approved the submitted version.

## Conflict of Interest

The authors declare that the research was conducted in the absence of any commercial or financial relationships that could be construed as a potential conflict of interest.
